# Oxygen-dependent functional brain haemodynamic response

**DOI:** 10.1364/BOE.545722

**Published:** 2025-03-17

**Authors:** Karolina Bejm, Stanislaw Wojtkiewicz, Zanna Pastuszak, Adam Liebert

**Affiliations:** 1 Nalecz Institute of Biocybernetics and Biomedical Engineering, Polish Academy of Sciences, Warsaw, Poland; 2 Laboratory of Preclinical Research and Environmental Agents, Mossakowski Medical Research Institute, Polish Academy of Sciences, Warsaw, Poland

## Abstract

The influence of hypoxia - a condition where tissues are under oxygen deficiency - on the human brain under functional load has not been fully understood yet. This study aims to analyse the effects of hypoxia on the brain’s haemodynamic response under visual stimulation, using the in-house developed functional near-infrared spectroscopy system and to quantify the hemodynamic response. Our results (median, 25^th^ and 75^th^ percentile) demonstrate the amplitude of the oxygenated haemoglobin functional response during hypoxia 0.30 µM (0.27, 0.41) was lower compared with the normoxia 0.63 µM (0.54, 0.93) and hyperoxia 0.73 µM (0.43, 1.09). No statistical significance is observed for the deoxygenated haemoglobin changes. The hypoxia has a statistically significant effect on the amplitude of the haemodynamic response (p < 0.001).

## 1. Introduction

A deficiency in oxygenation of a brain tissue (the hypoxia condition) may lead to a neuron damage. It emerges under many serious and life-threatening conditions like a heart attack or a stroke [1]. Furthermore, cerebral hypoxia results in diminished motor skills and coordination difficulties [2]. Hypoxia may take place climbing high altitudes and can lead to the acute mountain sickness [3]. Moreover, it is frequently observed in patient with COVID-19 [4–6]. Near infrared spectroscopy (NIRS) is a safe, non-invasive technique that measures light attenuation to monitor blood oxygenation changes [7]. It's portable, bedside-friendly, and compatible with implanted devices, making it an alternative to fMRI, EEG, or PET in certain cases. NIRS has been extensively tested in labs and clinics, showing promise in monitoring brain and muscle responses to stimulation, particularly useful for studying hypoxia [8–12].

A typical haemodynamic response to the hypoxia is an increase in the deoxygenated haemoglobin concentration and a decrease in the concentration of the oxygenated haemoglobin [13]. Lefferts et al. [14] investigated a moderate-intensity exercise under hypoxia with NIRS on prefrontal cortex (PFC) and the transcranial doppler ultrasonography of the middle cerebral artery. The oxygenation of the PFC increased during exercises under the normoxia and decreased during the same exercises carried out under the hypoxia. Similar pattern of changes in oxy- and deoxy-haemoglobin concentrations were observed in time-resolved NIRS measurements carried out at 14 wavelengths [15]. Davies et al [16] showed an ability of the NIRS technique to detect changes in cerebral tissue oxygenation during controlled hypoxia with both continuous wave and frequency domain NIRS monitors. Brain cortex oxygenation can also be monitored using NIRS in aviation pilots of highly maneuverable aircraft exposed to severe hypoxia, revealing decreased cerebral blood flow (CBF) in various brain regions. [17–19]. Effects of hypoxia were also investigated using the functional magnetic resonance imaging (fMRI) [20,21]. The fMRI studies of the visual cortex haemodynamic response to a mild hypoxia showed a significant decrease in the size of activation area in comparison to a normoxia condition.

The aim of this study is to investigate the hypoxia impact on a brain functional haemodynamic response to a typical task, i.e. a visual stimulation. We utilize in-house developed, high-density diffuse optical tomography (HD-DOT) continuous wave NIRS system. We will present changes in functional haemodynamic response within the visual cortex induced by the hypoxia condition. Further, we propose a method to parameterise the cortex response and to evaluate the visual response impairment caused by the hypoxia.

## 2. Materials and methods

### 2.1. Measurement system

**Fig. 1.**
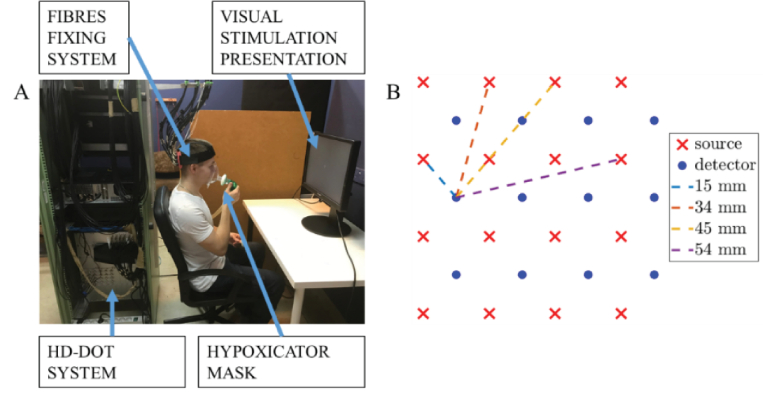
The measurement setup (A) and geometry of the fibres fixing system (B).

The cortical activity was recorded with in-house developed continuous wave high-density diffuse optical tomography setup HD-DOT [22]. Changes in the light intensity passing through the brain tissue were registered at all spatial combinations of 12 detectors and 16 sources at 2 wavelengths (192 source-detector pairs at 2 wavelengths – 750 nm and 850 nm). The imaging refresh rate for all pairs is 14.01 Hz. The LED light sources are modulated at kHz frequency range. The RMS power at the fibre tips does not exceed 5.2 mW at any of wavelengths used. Considering the 2.5 mm diameter of the active area at the source fibre bundle tip, the power density is 1.06 mW/mm^2^. Which satisfies the safe limit of 2 mW/mm^2^ for continuous tissue exposure (according to IEC 60601-2-57:2011). A set of 12 detecting and 16 source optical fibres was used to deliver and collect light at the head surface. A fixing system of the fibres was positioned symmetrically at the head surface above the occipital lobe. The hypoxicator system Go2Altitude (Biomedtech Australia Pty Ltd, Australia) was used to control the oxygen level and collect other data such as heart rate, respiratory rate and arterial saturation level. The air mixture was delivered with a dedicated breathing mask. For safety reasons, a subject holds the breathing mask itself. A pulse oximeter working with the hypoxicator was fixed on the index finger for monitoring the arterial saturation. The control software of the hypoxicator allows for starting in hypoxia mode, followed by hyperoxia mode. During normoxia, the hypoxicator was already operating in hypoxia mode, while the subject was breathing room air. Subject started the hypoxia condition putting the hypoxicator mask on. Consequently, the respiratory rate was not measured at the first normoxia stage. End-tidal CO_2_ was recorded with the use of a capnometer (CapnoTrue, UK) with its sensor mounted on the breathing mask. The measurement system is shown in Fig. 1.

We have developed a MATLAB-based software package for an on-line visualization of the high-density optical signals and their quality metrics. Furthermore, we have developed a software to generate a visual stimuli as reported in [23], display it to the subject and generate synchronization signal. A separate PC with a data acquisition card (National Instruments, USA) was used to register all synchronization signals within the system under a common sampling clock.

### 2.2. Subjects

A group of 10 healthy volunteers (4 men and 6 women) was examined. Age ranged from 28 to 42 years with the median of 33.5. Exclusion criteria were: hypertension, cerebrovascular and respiratory disorder, epilepsy and smoking. Measurements were carried out under a medical supervision. The experiment was approved by the Bioethics Committee at the Medical University of Warsaw.

### 2.3. Measurement protocol

**Fig. 2.**
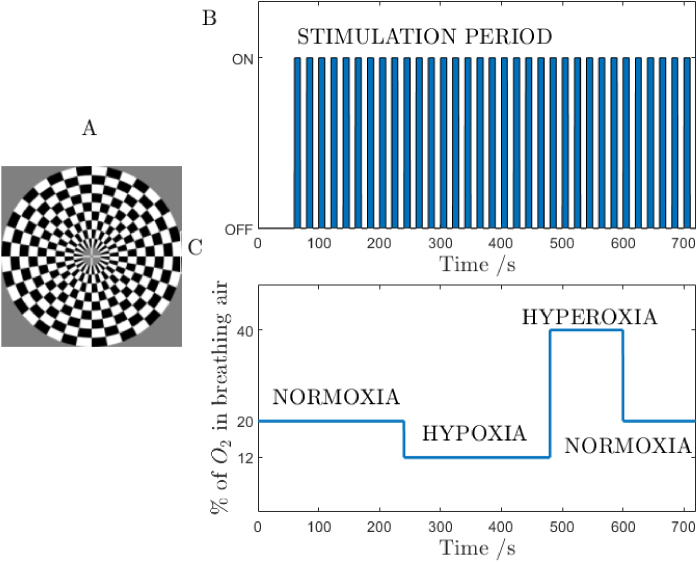
The measurement protocol. Panel (A) shows the visual cortex stimulation paradigm – the checkerboard. The train of visual stimulations is marked at panel (B). Panel (C) shows the oxygen concentration in the breathing air mixture throughout the measurement phases: normoxia, hypoxia, hyperoxia and a return to normoxia.

A subject was watching an image of a checkerboard blinking at a contrast reversing frequency of 8 Hz. A baseline of a rest period of 60 seconds is followed by 10-seconds stimulations separated by 10-seconds rest cycles as shown in Fig. 2. Measurements were carried out in a sitting position, with both eyes open. The subject’s head was positioned at the distance of 65 cm from a 20.1-inch screen operating with 4:3 ratio. Volunteers were informed to keep the visual fixation at white cross displayed at the centre of the screen. Comprehensive information on the visual stimulation is reported in [23]. The visual cortex activation was registered under three consecutive conditions: normal breathing (21% oxygen in the breathing air mixture), hypoxia challenge (12% oxygen) and hyperoxia (40% oxygen). Normoxia and hypoxia durations equal. However, there are more repetitions of the visual stimulation at the hypoxia as the normoxia includes the one-minute rest period. Our goal was set to observe changes of the response at the stimulation rate of 1/20 Hz.

### 2.4. Data analysis

Data analysis was carried out using the MATLAB environment. Initially, all signals available from all devices were synchronized. Signals acquired at different source-detector pairs were grouped based on available separations *r*_1_ = 15 mm, *r*_2_ = 34 mm, *r*_3_ = 45 mm and *r*_4_ = 54 mm defined by the fixing system geometry (see the geometry in Fig. 1).

Changes in intensity of the light passing through the tissue were converted into the haemoglobin concentrations changes using the modified Beer-Lambert law and the haemoglobin extinction coefficients taken from [24]. The optical pathlength was estimated as the product of the differential pathlength factor (DPF) and the selected source-detector separation (with DPF = 6 following [25]). All reported changes are related to a stationary part of the normoxia period at the beginning of the protocol. We do not introduce any regression operations on the large source-detector signals to remove the extracerebral information present at the 15 mm source-detector separation pairs. It was intended to minimise any influence to the low 1/20 Hz frequency of the visual stimulation paradigm. The goal was to avoid extensive signal processing before the frequency analysis.

Frequency analysis of non-averaged (non-conditioned) signals of the haemoglobin changes were carried out. Power density spectra were calculated using Fourier transform. A contrast to noise ratio (CNR) was calculated from normoxia period to indicate low quality source-detector measurement pairs, where no visual stimulation frequency of 1/20 Hz is present. The CNR was calculated as the amplitude of oxygenated haemoglobin at the visual stimulation frequency (1/20 Hz) normalized by the average of neighbouring frequencies amplitude. I.e. the average of three samples to the left and three samples to the right of the visual stimulus peak. Signals with the CNR below 1.5 were excluded from the analysis. E.g., the CNR rejection ratio for a representative subject was: 42 of 42 source-detector pairs (100%) at *r*_1_ = 15 mm, 44 of 58 pairs (76%) at *r*_2_ = 34 mm, 14 of 20 pairs (70%) at *r*_3_ = 45 mm and 28 of 32 pairs (88%) at *r*_4_ = 54 mm. The biggest number of pairs with observable visual stimulation was at the distance of *r*_2_ = 34 mm. As such, channels at *r*_2_ were used in further analysis. Only subjects with at least 10 valid channels at 34 mm source-detector distance were included in the analysis. Power density spectra for transition times between states (start of visual stimulation or the oxygen condition change) were neglected as contaminated with motion artefacts.

The next step was to extract from the power density spectra a time trace of the amplitude positioned at the visual stimulation frequency. I.e. the haemoglobin component oscillating at the visual stimulation response only. The frequency analysis was carried out within a sliding window. The window width was set to 1024 samples corresponding to 73 s. Therefore, on average, the window covers 3.65 visual stimulation epochs of 20 s long. The first 30 cycles of stimulation were analyzed. The hypoxicator provides heart rate (HR), respiratory rate (RR) and arterial saturation (SpO_2_). Additionally, the Pulse-Respiration Quotient (PRQ) parameter was calculated as a ratio of heart rate and respiratory rate for hypoxia and hyperoxia periods, as shown in [26].

A multivariate general linear model (GLM) was applied to check relations of measured parameters between experiment stages. Two GLM models were considered. One for changes between normoxia and hypoxia and the second one for hypoxia and hyperoxia: Following parameters were considered as responses: hart rate, respiratory rate, the PRQ and arterial saturation SpO_2_. The respiratory rate and PQR are available only for the hypoxia vs. hyperoxia model. Two predictors were set: corresponding changes in amplitude of visual stimulation response in oxygenated and deoxygenated haemoglobin.

### 2.5. Statistical data analysis

Changes in concentrations of oxy and deoxy haemoglobin at three conditions (normoxia, hypoxia and hyperoxia) were used as dependent variables. For all statistical analyses, p value of 0.05 was used as the cut off for significance. Normality of distributions and homogeneity of variances of the variables were checked using a Shapiro–Wilk test with a Lilliefors correction and Mauchley’s test. The condition of normal distribution was rejected. Therefore, the ANOVA Friedman’s test was applied for the model built of data from healthy volunteers, three conditions (normoxia, hypoxia, hyperoxia) and two chromophore concentrations (oxy and deoxy haemoglobin). The significance between changes in chromophores concentrations was tested using a Tuckey post-hoc test.

## 3. Results

**Fig. 3.**
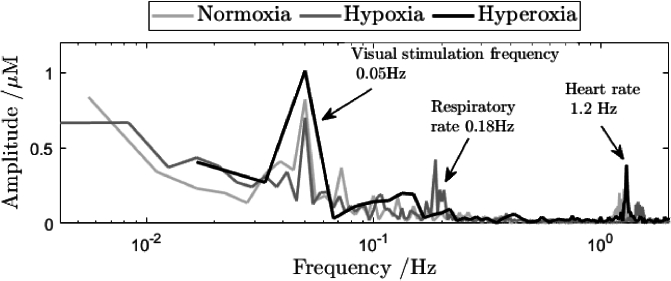
Power density spectra of oxygenated haemoglobin changes under normoxia (light grey line), hypoxia grey line) and hyperoxia (black line). Data for a representative source–detector pair at 34 mm separation.

Representative Fourier transforms of changes in oxygenated haemoglobin obtained during normoxia, hypoxia and hyperoxia are shown in Fig. 3. The spectrum is scaled to reveal amplitudes of the frequency components expressed in molars.

The first frequency peak on the left corresponds to the visual stimulation frequency (1/20 seconds) and is surrounded by noise. This peak is smallest during hypoxia, higher during normoxia, and reaches its highest value during hyperoxia. The second clearly visible peak represents the respiratory rate and is present only during hypoxia. The third peak indicates the heart rate and its location changes between experiment stages. The peak at around 0.07 Hz (not marked on Fig. 3) and visible in the normoxia is subject specific and may be considered an artefact An increase in the heart rate in the hypoxia shows the well-known control mechanism of compensating for the lack of blood oxygen saturation by the increase in the blood flow. Data in the time domain as observed in the proposed protocol are shown in Fig. 5.

**Fig. 4.**
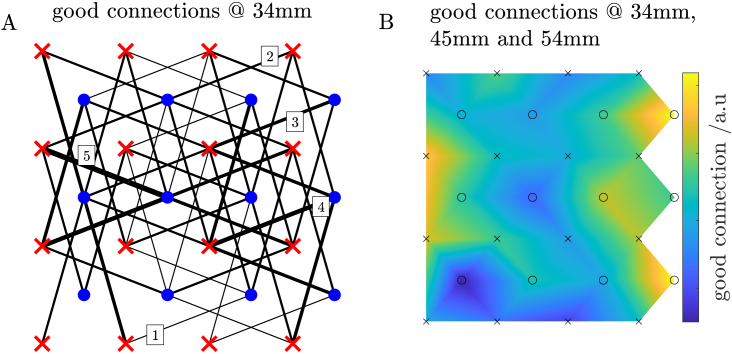
Spatial distribution of source-detector pairs with good contrast to noise ratio (CNR) across all subjects for the second nearest neighbors (A) and for all channels passing the CNR condition (B). Source-detector layout corresponds to Fig. 1(B). Lines’ widths and numbers (from 1 to 5) on panel A indicate how many times given source-detector pair had good CNR within the group of all subjects.

Figure 4 shows spatial distribution of source-detector pairs with good SNR across all subjects. Source-detector layout corresponds to Fig. 1(B).

**Fig. 5.**
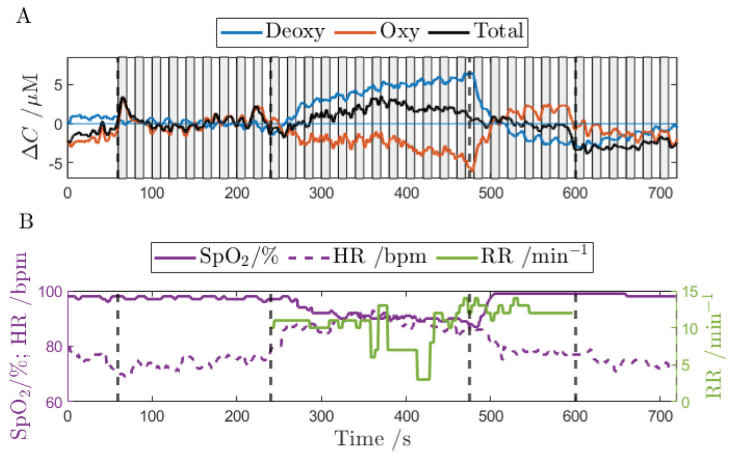
Representative signals acquired at source-detector distance of 34 mm. (A) changes in haemoglobin Oxy – oxygenated, Deoxy – deoxygenated and Total = Oxy + Deoxy. Grey vertical bars in panel (A) highlight the visual stimulation periods. (B) arterial oxygen saturation (SpO_2_), respiratory rate (RR), and heart rate (HR) measured with the hypoxicator. The respiratory rate is present only when the subject is breathing through the mask. Vertical dashed lines indicate the experimental stages corresponding to Fig. 2(C).

All observed parameters appear stable at the normoxia state. Furthermore, a gradual decrease within the hypoxia state is observed in: the arterial saturation and the amplitude of haemodynamic response to the visual stimulation. The decrease is visible for both haemoglobin forms: oxygenated and deoxygenated haemoglobin. The respiratory rate tends to slow down in the hypoxia as some subjects started to make longer and deeper breaths. In some subjects the respiratory rate remained stable throughout the experiment. In preliminary measurements carried out on 3 subjects, no evident changes in end-tidal CO_2_ were observed. The capnometer connected to the mask showed around 40 mmHg during the mask-breathing phase. It shows the volunteers were able to breathe with no obstructions. Values for the whole group of volunteers are presented as the median, 25^th^ and 75^th^ percentiles as follows: heart rate (HR) was highest during hypoxia 92.65 bpm (89.33, 99.87) compared to both hyperoxia 80.51 bpm (77.54, 81.78) and normoxia 79.25 bpm (79.01, 81.49). Only slight changes in respiratory rate (RR) were observed between hypoxia 10.89 min^−1^ (10.17, 11.72) and hyperoxia 12.14 min^−1^ (11.83, 13.43). Based on the available data, the PRQ parameter was calculated, revealing higher PRQ values during hypoxia 9.12 (7.91, 9.65) compared to hyperoxia 6.61 (5.94, 7.25). SpO_2_ levels were lowest during hypoxia 86.54% (85.86, 87.57) and remained similar between normoxia 97.81% (97.16, 98) and hyperoxia 94.93% (92.23, 95.58).

The GLM model for changes between normoxia and hypoxia can predict heart rate with 10% error and arterial saturation with 7% error, considering changes in amplitude of visual stimulation response in oxygenated and deoxygenated haemoglobin as predictors. For the model of hypoxia followed by hyperoxia, the following can be predicted: heart rate (5% error), respiratory rate (75% error), Pulse-Respiration Quotient (41% error) and arterial saturation (7% error). Therefore, the prediction does not work well for the parameters related to the respiratory rate.

**Fig. 6.**
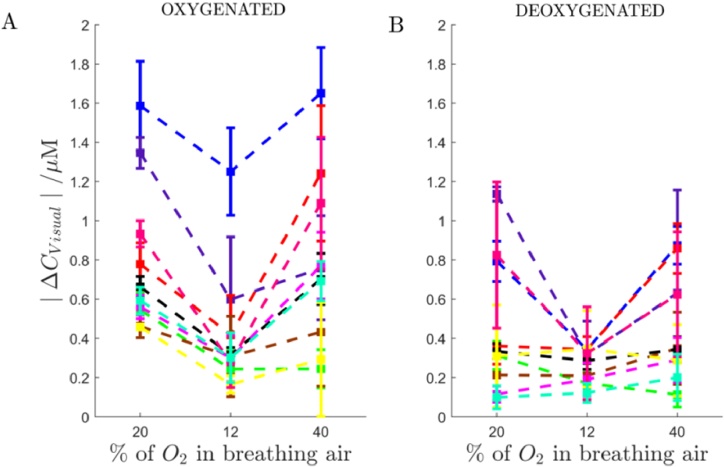
Amplitudes of changes in (A) oxygenated and (B) deoxygenated haemoglobin concentrations oscillating at the visual stimulation frequency (0.05 Hz) as a function of various oxygen level in the breathing gas mixture (20% oxygen concentration for normoxia, 12% oxygen concentration for hypoxia and 40% oxygen concentration for hyperoxia). Data shown for source-detector pairs at the distance of 34 mm. The colours correspond to subjects (group size *n* = 10- including 3 rejected subjects). Each coloured point represents the averaged value for each experiment period, calculated for each subject from all pairs that qualified for the analysis. Standard deviations show variability between source-detector pairs.

Figure 6 shows the amplitude of changes in the haemodynamic response to the visual stimulation as a function of the oxygen content within the breathing air. The |Δ*C*_Visual_| is the amplitude of the frequency component oscillating at the visual stimulation frequency of 0.05 Hz.

**Fig. 7.**
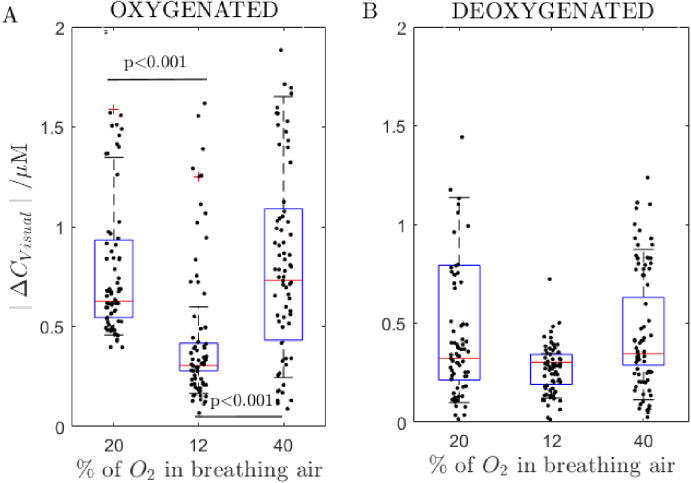
(A) Distributions of amplitudes of the haemodynamic response to the visual stimulation at varying content of oxygen in breathing air for 7 healthy subjects: oxygenated haemoglobin, (B) deoxygenated haemoglobin. Normoxia (20% O_2_), hypoxia (12% O_2_) and hyperoxia (40% O_2_). Central red line within a box marks the median, the bottom and top box boundaries correspond to 25th and 75th percentiles respectively. The whiskers show data range and red ‘+’ marks outliers. The statistical significance is marked with the *p* value. The black dots represent values for all source-detector separations qualified for the analysis for all subjects (10 pairs with the highest amplitude from each of the 7 subject).

Figure 7 shows distributions of amplitudes of the haemodynamic response to the visual stimulation at varying content of oxygen in breathing air for 7 healthy subjects (distributions of data from Fig. 6). The highest amplitude of the haemodynamic response to the visual stimulation is observed at the hyperoxia (the 40% O_2_ concentration). The lowest value is at the hypoxia (for 12% of O_2_in the mixture).

In 3 out of 10 subjects, insufficient number of channels with the CNR of 1.5 was registered. Therefore, for technical reasons data from 3 subjects was excluded from the further statistical analysis.

Results show that the amplitude of the haemodynamic response to the visual stimulation is statistically different between hypoxia and normoxia or hypoxia and hyperoxia. No statistical significance is observed for the changes in deoxygenated haemoglobin observed at the frequency of the visual stimulation. The medians and 25^th^ and 75^th^ percentiles as in Fig. 7 are: for oxygenated haemoglobin 0.63 µM (0.54, 0.93) normoxia, 0.30 µM (0.27, 0.41) hypoxia, and 0.73 µM (0.43, 1.01) hyperoxia and for deoxygenated haemoglobin 0.32µM (0.21, 0.79) normoxia, 0.30 µM (0.19, 0.34) hypoxia and 0.35 µM (0.29, 0.63).

**Fig. 8.**
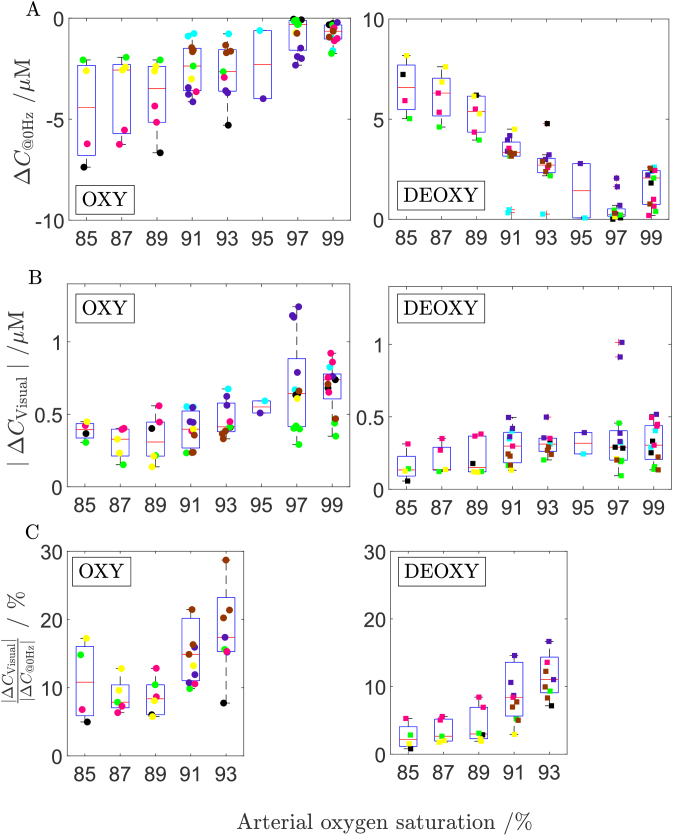
(A)- The haemodynamic response at the non-fluctuating components of the signals of oxy- and deoxyhaemoglobin changes (ΔC_@0Hz_) related to the arterial saturation SpO_2_ (B)-Amplitude of the haemodynamic response at the visual stimulation frequency of 0.05 Hz vs. the arterial oxygen saturation. (C)- Ratio of the haemodynamic responses at the visual stimulation |Δ*C*_Visual_| to the non-fluctuating component (ΔC_@0Hz_) - (C). Colours correspond to the 7 subjects. Data registered at the source-detector separation of 34 mm. Circles correspond to changes in oxygenated haemoglobin, whereas rectangles indicate changes in deoxygenated haemoglobin. Data (markers) shown together with its distributions (boxplots).

Figure 8 A shows haemodynamic response at the non-fluctuating components of the signals of oxy- and deoxyghaemoglobin changes (Δ*C*_@0Hz_) related to the changes in the arterial saturation SpO_2_. This result refers to the systemic, non-functional haemodynamic response which is not related to the visual stimulation. The typical ‘scissors’ shape [27] can be observed - concentration of oxygenated haemoglobin decreases and deoxygenated increases with the decrease of arterial saturation. Figure 8 B shows the relation between the haemodynamic response at the visual stimulation frequency (Δ*C*_Visual_) and the arterial saturation SpO_2_.

Results presented in Fig. 8 reflect the data presented in Fig. 7, however the haemodynamic response is shown not in the relation to the percentage of the oxygen in breathing air but to the arterial oxygen saturation. The amplitude of change at the visual frequency decreases gradually with the arterial saturation level for concentrations of both haemoglobins. The highest amplitude of visual response is observed for hyperoxia (arterial saturation level – 99%) whereas the lowest value is observed for hypoxia (arterial saturation level range 85-89%).

Figure 8(C) presents the ratio of the haemodynamic response at the visual stimulation |Δ*C*_Visual_| to the non-fluctuating component (Δ*C*_@0Hz_) not related to the visual stimulation. This ratio is shown in relation to the arterial saturation.

The ratio as shown in Fig. 8 C decreases gradually with the arterial saturation level. The highest value is observed for the normoxia (arterial saturation level starting at – 91-93%), whereas the lowest value is observed for hypoxia (arterial saturation level ranges around 85-89%). The proposed parameter (the ratio) is undefined for high saturation level (SpO_2_ > 95%) as the non-fluctuating components (Δ*C*__@0Hz__) in the denominator do not change and approach zero.

**Fig. 9.**
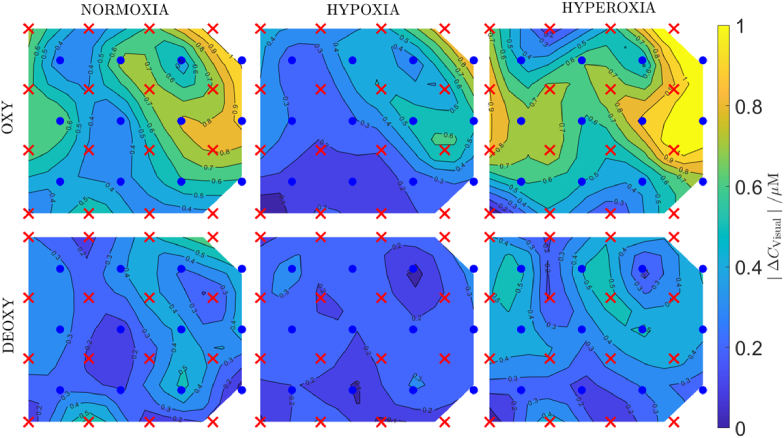
Spatial distributions of the visual cortex haemodynamic response, averaged for all qualified subjects (7 subjects, 10 source-detector pairs each). Colours correspond to the amplitude of the visual stimulation (at 0.05 Hz). The geometry corresponds to the fibres fixing system as in Fig. 1(B). Data registered at the 34 mm source-detector separation.

A topographic map of the visual cortex haemodynamic response as used in the statistical analysis, averaged for all qualified subjects (7 subjects, 10 source-detector pairs each) is shown in Fig. 9. Data is registered at the 34 mm source-detector separation. Images correspond to the fibres fixing geometry as shown in Fig. 1(B) The local concentration of oxygenated haemoglobin increased in the visual cortex area during hyperoxia, associated with an increase in amplitude of the stimulus-related changes at visual stimulation frequency. The opposite effect is observed under hypoxia, where amplitude and size of the activated area decreases. The similar trend is visible for deoxygenated haemoglobin. However, the amplitudes are lower.

## 4. Discussion

The use of the high dynamic range HD-DOT functional NIRS system enabled imaging of frequency components of the visual cortex haemodynamic response. Results show the oxygen level in inhaled air has a significant effect on the amplitude of the brain haemodynamic response to a visual stimulus. The haemoglobin concentration changes observed in the frequency domain revealed statistically significant drop in the amplitude of the oxygenated haemoglobin changes at the visual stimulation frequency during the hypoxia. Furthermore, the hyperoxia increased the response amplitude to its highest observed level. However, this effect might be amplified by the recovery from hypoxia to hyperoxia. Due to the limitations in the experimental setup, we were unable to fully investigate this effect.

No significant difference between the left and right hemispheres are expected, as both eyes were stimulated equally during the experiment. Our previous study [23] carried out with the same HD-DOT device and visual stimulation protocol show only minor differences between sides (left and right hemisphere) in amplitudes of oxy- and deoxy hemoglobin changes during normoxia. Some left-right difference can be observed in Fig. 9.

We noted typical opposite directions of changes in oxygenated and deoxygenated haemoglobin as a response to the decrease in the arterial oxygen saturation. Similar response has been reported by Gerega et al. for a hypoxic challenge conditions [15]. We notice a 50% drop in the amplitude of the functional haemodynamic response of the visual cortex when the oxygen content in the breathing air drops from 20% to 12% (refer to Fig. 7). Other authors reported the brain haemodynamic response decrease during a mild hypoxia [28,29]. In the study by Ho et al. it was shown the vascular reactivity is not affected by a mild hypoxia (for inspired oxygen mixture higher than 85%) and a decrease in number of activated brain voxels in the fMRI technique was observed [21].

The variance of the visual stimulation response changes significantly between normoxia, hypoxia and hyperoxia. This observation is inter-subject as shown in Fig. 6, where subjects exhibit different responses following hypoxia. We hypothesize this variability may represent individual-specific recovery mechanisms after oxygen deprivation. Differences in the variance could follow unique vascular and metabolic responses in each subject during the rapid transition from low oxygen (hypoxia) to high oxygen (hyperoxia) or normoxia. Results as published in [30] might support this thesis, where significant variations in haematological responses to bed rest and hypoxia were observed among subjects. The researchers noted that this variability in haematological variables is primarily driven by mechanisms responding to hypoxic acclimation rather than inactivity alone

In this work we do not register changes in cerebral blood flow (CBF) directly. However, we observe a slight increase in the total haemoglobin during the hypoxia which may be associated with increase in blood content in the stimulated cortex area. In several studies it was shown that reduced or even completely abolished BOLD response is noted during a severe hypoxia (SpO_2_ < 80%) with a significant change in the CBF [21,31,32]. However, results of these studies cannot be directly compared to our findings due to differences in responses to very low oxygen content in the inspired gas mixture. Hypoxic conditions impair haemodynamic reactions in various regions of the brain cortex, as demonstrated by a study on rats exposed to acute hypoxia, revealing a reduced cochlear replay at high frequencies. [33].

The observed increase in the amplitude of haemodynamic response in the hyperoxia matches well with results presented in [34], where the CBF and the BOLD signals were investigated. Authors reported that the CBF increases under the hyperoxia and the amplitude of the BOLD signal related to a visual stimulation increases as well.

The deoxy-haemoglobin concentration significantly increases at the systemic level in response to the hypoxia, i.e. at the 0 Hz (the haemoglobin ‘scissors’ pattern observed in Fig. 5(a) and 8). This increase shows the systemic response to hypoxia where oxygen is consumed at higher pace than it is replenished. However, our results show the statistically insignificant change in the amplitude of deoxy-haemoglobin concentration induced by the visual stimulation. This suggests, the local cortex response to the visual stimulation and systemic hypoxia response do not show the same pattern.

Increase in the heart rate during hypoxia is a common physiological phenomena – more blood is pumped by the heart to compensate for the decrease in oxygen supply [35]. The constant level of the end-tidal CO_2_ during the hypoxic challenge suggests the CBF was rather stable during the experiment [36]. All participants reacted similarly to changes in the oxygen concentration of the inspired gas mixture. However, the amplitudes of the responses to visual stimulation |Δ*C*_Visual_| varied between individuals. Further, the respiratory rate (RR) frequency component in the NIRS signal was observed only during the hypoxia period. This can be attributed to the body's natural hypoxic ventilatory response (HVR), which increases breathing to improve oxygen intake and remove carbon dioxide [37]. Studies have shown that extracting the RR from NIRS signals is challenging. However, algorithms developed for cerebral NIRS and fNIRS data highlight both the potential and challenges of using these signals for respiratory monitoring, emphasizing the complexity of the RR extraction and the need for refined methods [38,39].

We propose the parameter for quantifying the effect of hypoxia on brain tissue. The ratio of the non-fluctuating component (Δ*C*__@0Hz__) to the haemodynamic response during visual stimulation |Δ*C*_Visual_| are presented as a function of arterial oxygen saturation. The proposed parameter could potentially be used to determine a critical brain cortex saturation level at which the functional reaction cannot be observed. The assessment of the critical saturation level could help to differentiate brain cortex reactivity and sensitivity to hypoxia among various groups of subjects [40] or patients considering their physical condition and physical activity level [41].

The decrease in visual cortex response during the hypoxia confirms that a lack of oxygen leads to generalized impairment of cognitive functions. Measurements as reported in [42] during exercise under hypoxia, showed a strong correlation between the increase in premotor time and decrease in the cerebral oxygenation. Furthermore, it was shown that a prolonged exercise under hypoxic conditions leads to a reduction in the cerebral oxygenation and significantly influences cognitive functions [43–45]. Studies carried out in aviation pilots showed that the hypoxia leads to highly impaired mean error frequency in memory tests [46] and an impairment of a standing balance [47].

## 5. Conclusions

We have demonstrated and quantified the influence of hypoxia on the cerebral functional haemodynamic response in the visual cortex. A significant impairment of the visual cortex haemodynamic response reaching 50% decrease was observed when the oxygen saturation was reduced to 85%. It is expected that this reduction in the amplitude of the cerebral haemodynamic reaction will persist with prolonged hypoxia or under lower inspired oxygen concentrations. The dynamics of the brain cortex response to the insufficient oxygen levels in the inspired gas mixture are subject-specific. Consequently, it is reasonable to anticipate that the progression of cognitive function loss is also individual-specific and linked to arterial oxygen saturation (SpO_2_). Calibrating this relationship on a subject-specific basis could potentially allow for the evaluation of individual responsiveness to low oxygen conditions. This has the potential to be valuable in monitoring critical personnel or individuals exposed to extreme conditions.

## Data Availability

Data underlying the results presented in this paper are not publicly available at this time but may be obtained from the authors upon reasonable request.

## References

[1] Ferdinand P. Roffe C., “Hypoxia after stroke: a review of experimental and clinical evidence,” Exp. & Trans. Stroke Med. 8(1), 9 (2016). 10.1186/s13231-016-0023-0 PMC5143450 27980710

[2] Ando S. Hatamoto Y. Sudo M. et al., “The effects of exercise under hypoxia on cognitive function,” PLoS One 8(5), e63630 (2013). 10.1371/journal.pone.0063630 23675496 PMC3651238

[3] Roach R. C. Hackett P. H., “Frontiers of hypoxia research: acute mountain sickness,” J. Exp. Biol. 204(18), 3161–3170 (2001). 10.1242/jeb.204.18.3161 11581330

[4] Guo Y.-R. Cao Q.-D. Hong Z.-S. et al., “The origin, transmission and clinical therapies on coronavirus disease 2019 (COVID-19) outbreak–an update on the status,” Mil. Med. Res. 7(1), 11 (2020). 10.1186/s40779-020-00240-0 32169119 PMC7068984

[5] Simonson T. S. Baker T. L. Banzett R. B. et al., “Silent hypoxaemia in COVID-19 patients,” J. Physiol. 599(4), 1057–1065 (2021). 10.1113/JP280769 33347610 PMC7902403

[6] Sirohiya P. Elavarasi A. Sagiraju H. K. R. et al., “Silent Hypoxia in Coronavirus disease-2019: Is it more dangerous? A retrospective cohort study,” Lung India 39(3), 247–253 (2022). 10.4103/lungindia.lungindia_601_21 35488682 PMC9200195

[7] Jobsis F. F., “Noninvasive, infrared monitoring of cerebral and myocardial oxygen sufficiency and circulatory parameters,” Science 198(4323), 1264–1267 (1977). 10.1126/science.929199 929199

[8] Pinti P. Tachtsidis I. Hamilton A. et al., “The present and future use of functional near-infrared spectroscopy (fNIRS) for cognitive neuroscience,” Ann. N. Y. Acad. Sci. 1464(1), 5–29 (2020). 10.1111/nyas.13948 30085354 PMC6367070

[9] Quaresima V. Ferrari M., “Functional near-infrared spectroscopy (fNIRS) for assessing cerebral cortex function during human behavior in natural/social situations: a concise review,” Organizational Res. Methods 22(1), 46–68 (2019). 10.1177/1094428116658959

[10] Sawosz P. Kacprzak M. Pulawski P. et al., “Influence of intra-abdominal pressure on the amplitude of fluctuations of cerebral hemoglobin concentration in the respiratory band,” Biomed. Opt. Express 10(7), 3434–3446 (2019). 10.1364/BOE.10.003434 31467788 PMC6706036

[11] Herold F. Wiegel P. Scholkmann F. et al., “Applications of functional near-infrared spectroscopy (fNIRS) neuroimaging in exercise-cognition science: a systematic, methodology-focused review,” J. Clin. Med. 7(12), 466 (2018). 10.3390/jcm7120466 30469482 PMC6306799

[12] Fantini S. Frederick B. Sassaroli A., “Perspective: prospects of non-invasive sensing of the human brain with diffuse optical imaging,” APL Photonics 3(11), 110901 (2018). 10.1063/1.5038571 31187064 PMC6559748

[13] Bale G. Elwell C. E. Tachtsidis I., “From Jöbsis to the present day: a review of clinical near-infrared spectroscopy measurements of cerebral cytochrome-c-oxidase,” J. Biomed. Opt. 21(9), 091307 (2016). 10.1117/1.JBO.21.9.091307 27170072

[14] Lefferts W. K. Babcock M. C. Tiss M. J. et al., “Effect of hypoxia on cerebrovascular and cognitive function during moderate intensity exercise,” Physiol. Behav. 165, 108–118 (2016). 10.1016/j.physbeh.2016.07.003 27402021

[15] Gerega A. Weigl W. Milej D. et al., “Multiwavelength time-resolved measurement of diffuse reflectance for brain oxygenation assessment during hypoxic challenge test,” in *Optical Molecular Probes, Imaging and Drug Delivery* (Optical Society of America, 2011).

[16] Davies D. J. Clancy M. Lighter D. et al., “Frequency-domain vs continuous-wave near-infrared spectroscopy devices: a comparison of clinically viable monitors in controlled hypoxia,” J. Clin. Monit. Comput. 31(5), 967–974 (2017). 10.1007/s10877-016-9942-5 27778208 PMC5599440

[17] Liu J. Li S. Qian L. et al., “Effects of acute mild hypoxia on cerebral blood flow in pilots,” Neurol. Sci. 42(2), 673–680 (2021). 10.1007/s10072-020-04567-3 32654008

[18] Gerega A. Wojtkiewicz S. Sawosz P. et al., “Assessment of the brain ischemia during orthostatic stress and lower body negative pressure in air force pilots by near-infrared spectroscopy,” Biomed. Opt. Express 11(2), 1043–1060 (2020). 10.1364/BOE.377779 32133236 PMC7041453

[19] Uchida K. Baker S. E. Wiggins C. C. et al., “Relationship between decreased oxygenation during acute hypoxia and cognitive deterioration in healthy humans,” The FASEB J. 34(S1), 1 (2020). 10.1096/fasebj.2020.34.s1.01944

[20] Barreto R. Mangia F. S. Garrido Salmon C. E., “Effects of reduced oxygen availability on the vascular response and oxygen consumption of the activated human visual cortex,” J. Magn. Reson. Imaging 46(1), 142–149 (2017). 10.1002/jmri.25537 27807911

[21] Ho Y.-C. L. Vidyasagar R. Shen Y. et al., “The BOLD response and vascular reactivity during visual stimulation in the presence of hypoxic hypoxia,” NeuroImage 41(2), 179–188 (2008). 10.1016/j.neuroimage.2008.02.048 18396415

[22] Wojtkiewicz S. Sawosz P. Kacprzak M. et al., “Towards optical tomography of an adult human head,” in *Optical Tomography and Spectroscopy* (Optical Society of America, 2016).

[23] Bejm K. Wojtkiewicz S. Sawosz P. et al., “Influence of contrast-reversing frequency on the amplitude and spatial distribution of visual cortex hemodynamic responses,” Biomed. Opt. Express 10(12), 6296–6312 (2019). 10.1364/BOE.10.006296 31853401 PMC6913388

[24] Prahl S., “Tabulated molar extinction coefficient for hemoglobin in water,” http://omlc.ogi.edu/spectra/hemoglobin/summary.html, 1999.

[25] Duncan A. Meek J. H. Clemence M. et al., “Optical pathlength measurements on adult head, calf and forearm and the head of the newborn infant using phase resolved optical spectroscopy,” Phys. Med. Biol. 40(2), 295–304 (1995). 10.1088/0031-9155/40/2/007 7708855

[26] Scholkmann F. Wolf U., “The pulse-respiration quotient: A powerful but untapped parameter for modern studies about human physiology and pathophysiology,” Front. Physiol. 10, 371 (2019). 10.3389/fphys.2019.00371 31024336 PMC6465339

[27] Bennet L. Peebles D. M. Edwards A. D. et al., “The cerebral hemodynamic response to asphyxia and hypoxia in the near-term fetal sheep as measured by near infrared spectroscopy,” Pediatr. Res. 44(6), 951–957 (1998). 10.1203/00006450-199812000-00022 9853934

[28] Tuunanen P. I. Kauppinen R. A., “Effects of oxygen saturation on BOLD and arterial spin labelling perfusion fMRI signals studied in a motor activation task,” NeuroImage 30(1), 102–109 (2006). 10.1016/j.neuroimage.2005.09.021 16243545

[29] Mintun M. A. Lundstrom B. N. Snyder A. Z. et al., “Blood flow and oxygen delivery to human brain during functional activity: theoretical modeling and experimental data,” Proc. Natl. Acad. Sci. 98(12), 6859–6864 (2001). 10.1073/pnas.111164398 11381119 PMC34443

[30] Royal J. T. Eiken O. Keramidas M. E. et al., “Heterogeneity of Hematological Response to Hypoxia and Short-Term or Medium-Term Bed Rest,” Front. Physiol. 12, 777611 (2021). 10.3389/fphys.2021.777611 34975531 PMC8715762

[31] Rostrup E. Larsson H. B.W. Born A. P. et al., “Changes in BOLD and ADC weighted imaging in acute hypoxia during sea-level and altitude adapted states,” NeuroImage 28(4), 947–955 (2005). 10.1016/j.neuroimage.2005.06.032 16095921

[32] Shen Y. Ho Y.-C. L. Vidyasagar R. et al., “Gray matter nulled and vascular space occupancy dependent fMRI response to visual stimulation during hypoxic hypoxia,” NeuroImage 59(4), 3450–3456 (2012). 10.1016/j.neuroimage.2011.10.097 22079453

[33] Cicek M. T. Koca C. F. Akarcay M., “The effects of acute hypoxia on audition: An experimental study,” North Clin. İstanbul 8(1), 1–7 (2020). 10.14744/nci.2020.10586 PMC7881432 33623866

[34] Kashikura K. Kershaw J. Kashikura A. et al., “Hyperoxia-enhanced activation-induced hemodynamic response in human VI: an fMRI study,” Neuroreport 11(5), 903–906 (2000). 10.1097/00001756-200004070-00001 10790852

[35] Talbot N. P. Balanos G. M. Dorrington K. L. et al., “Two temporal components within the human pulmonary vascular response to∼ 2 h of isocapnic hypoxia,” J. Appl. Physiol. 98(3), 1125–1139 (2005). 10.1152/japplphysiol.00903.2004 15542574

[36] Søvik S. Lossius K., “Development of ventilatory response to transient hypercapnia and hypercapnic hypoxia in term infants,” Pediatr. Res. 55(2), 302–309 (2004). 10.1203/01.PDR.0000106316.40213.DB 14630982

[37] Oeung B. Pham K. Olfert I. M. et al., “The normal distribution of the hypoxic ventilatory response and methodological impacts: A meta-analysis and computational investigation,” The J. Physiol. 601(19), 4423–4440 (2023). 10.1113/JP284767 37589511 PMC10543592

[38] Hakimi N. Shahbakhti M. Sappia S. et al., “Estimation of respiratory rate from functional near-infrared spectroscopy (fNIRS): a new perspective on respiratory interference,” Biosensors 12(12), 1170 (2022). 10.3390/bios12121170 36551137 PMC9775029

[39] Hakimi N. Shahbakhti M. Horschig J. M. et al., “Respiratory rate extraction from neonatal near-infrared spectroscopy signals,” Sensors 23(9), 4487 (2023). 10.3390/s23094487 37177691 PMC10181728

[40] Mourot L. Millet G. P., “Is Maximal Heart Rate Decrease Similar Between Normobaric Versus Hypobaric Hypoxia in Trained and Untrained Subjects?” High Alt. Med. Biol. 20(1), 94–98 (2019). 10.1089/ham.2018.0104 30489174

[41] Goenarjo R. Bosquet L. Berryman N. et al., “Cerebral Oxygenation Reserve: The Relationship Between Physical Activity Level and the Cognitive Load During a Stroop Task in Healthy Young Males,” Int. J. Environ. Res. Public Health 17(4), 1406 (2020). 10.3390/ijerph17041406 32098221 PMC7068614

[42] Ando S. Yamada Y. Kokubu M., “Reaction time to peripheral visual stimuli during exercise under hypoxia,” J. Appl. Physiol. 108(5), 1210–1216 (2010). 10.1152/japplphysiol.01115.2009 20167674

[43] Dobashi S. Horiuchi M. Endo J. et al., “Cognitive function and cerebral oxygenation during prolonged exercise under hypoxia in healthy young males,” High Altitude Med. Bio. 17(3), 214–221 (2016). 10.1089/ham.2016.0036 27584683

[44] Marillier M. Rupp T. Bouzat P. et al., “Cerebral haemodynamics and oxygenation during whole-body exercise over 5 days at high altitude,” Exp. Physiol. 106(1), 65–75 (2021). 10.1113/EP088354 31999870

[45] Salgado R. M. Coffman K. E. Bradbury K. E. et al., “Effect of 8 days of exercise-heat acclimation on aerobic exercise performance of men in hypobaric hypoxia,” Am. J. Physiol. Regul. Integr. Comp. Physiol. 319(1), R114–R122 (2020). 10.1152/ajpregu.00048.2020 32432914

[46] Malle C. Quinette P. Laisney M. et al., “Working memory impairment in pilots exposed to acute hypobaric hypoxia,” Aviation, Space, Environ. Med. 84(8), 773–779 (2013). 10.3357/ASEM.3482.2013 23926651

[47] Debenham M. I. B. Smuin J. N. Grantham T. D. A. et al., “Hypoxia and standing balance,” Eur. J. Appl. Physiol. 121(4), 993–1008 (2021). 10.1007/s00421-020-04581-5 33484334

